# Culture of Cultures: A Small Step Towards Augmenting Diagnostic Stewardship

**DOI:** 10.7759/cureus.63451

**Published:** 2024-06-29

**Authors:** Shashank Cheemalapati, Deepashree R, Sujatha S R, Krishna Karthik M V S, Narayanappa D

**Affiliations:** 1 Department of General Medicine, JSS Medical College and Hospital, Mysore, IND; 2 Department of Microbiology, JSS Medical College and Hospital, Mysore, IND; 3 Department of Paediatrics, JSS Medical College and Hospital, Mysore, IND

**Keywords:** antimicrobial stewardship practice, antimicrobial prescribing practices, antimicrobial surveillance, antimicrobial stewardship program, adherence, antimicrobial resistance

## Abstract

Laboratories with a well-established diagnostic stewardship program for culture and the antimicrobial susceptibility test (C-AST) play a key role in guiding clinicians to institute specific targeted therapy. Blood culture is one of the most critical investigations performed at a microbiology laboratory. Therefore, it is particularly important to develop a robust diagnostic stewardship model for the blood culture laboratory division. Aiming at this hypothesis, this hospital-based clinical audit carried out within the Department of General Medicine, centered on the critical domain of antimicrobial stewardship (AMSP). The audit's primary objective was to systematically evaluate prevailing practices and pinpoint areas necessitating refinement in the administration of antimicrobial agents. Employing a meticulous approach involving exhaustive data scrutiny and feedback mechanisms, the audit unearthed strategic opportunities to optimize prescription patterns, curtail unwarranted antimicrobial utilization, and fortify adherence to established guidelines. The subsequent execution of targeted interventions, encompassing educational initiatives and routine performance feedback, culminated in a noteworthy enhancement of antimicrobial prescribing practices. These outcomes unequivocally underscore the efficacy of the audit in cultivating a milieu of judicious antimicrobial utilization, thereby augmenting patient care and mitigating antibiotic resistance within the department.

## Introduction

Antimicrobial resistance (AMR) is now an emerging threat globally, which is not only influencing public health but also posing a significant problem for economic development and security. When looking into the global scenario, AMR can be a potential cause of over 10 million deaths by the end of 2050 [1]. This threat has highlighted the urgent and unprecedented need for antimicrobial stewardship (AMSP). The World Bank has also declared that there can be a dire rise in healthcare costs that can go up to one trillion US dollars by 2050 due to AMR [2]. There are various ways of implementing AMSP, and one of the important backbones for most of the strategies is having a microbiological culture sensitivity report [3]. In most low- and middle-income countries (LMICs), physicians’ antibiotic treatment prescriptions are largely empirical and broad-spectrum rather than achieving pathogen-specific treatment [4]. When it comes to cost reduction in diagnosis and treatment, the majority of physicians opt for administering multiple or broad-spectrum antimicrobial agents rather than initiating treatment as per the recommended guidelines, along with sending for microbiological culture investigations. During AMSP rounds, in the absence of culture investigations, critical actions such as escalation and de-escalation are challenging to execute with a solid evidential basis. Instead, these decisions are often made based on the patient's clinical improvement [5]. Microbial culture investigations are essential in AMSP by pinpointing the specific pathogens causing infections and their antibiotic susceptibility. This precise information enables healthcare providers to tailor antibiotic treatment, preventing overuse of broad-spectrum drugs, minimizing antibiotic resistance, and reducing adverse effects [6]. It guides optimal antibiotic selection and treatment duration, lowering healthcare costs. Additionally, culture results aid in tracking resistance patterns, educating both providers and patients about appropriate antibiotic use, and ensuring the right treatment for the right patient [7].

Antimicrobial resistance triggers due to irrational usage, either by overuse or misuse of antimicrobial agents [8]. Trends of antimicrobial misuse in hospitals, including failure to de-boost and overprescription of broad-spectrum antibiotics, remained unchanged at 50%. This also included failure to escalate and de-escalate the antimicrobial agents whenever needed. Hence, AMSP applications (ASPs) are one way to cope with inappropriate antimicrobial use and AMR. The main goal of these ASPs is to mainly concentrate on improving patient outcomes and safety, which in turn reduces the burden of AMR, and healthcare costs that promote rationale usage of antibiotics. A few of the core elements noted in successful ASPs constitute the commitment of the leaders, accountability by the prescribers, expertise in the field, and proper education of the healthcare workers (HCWs), including patients and stakeholders. [9,10].

In summary, microbial culture investigations are indispensable for effective, targeted, and responsible antibiotic management, improving patient outcomes, and combating AMR. Hence, this study was undertaken to understand the practice of sending microbial cultures before starting antimicrobial agents and also to design an intervention highlighting the importance of the correlation between the microbial culture sent and the culture positivity rate. Also, an effort was made to correlate the compliance of empirical treatment prescribed according to hospital antibiotic policy.

## Materials and methods

Type of study

This was an interventional study.

Study setting

This interventional study was conducted at JSS Medical College and Hospital, a tertiary care hospital situated in Mysore, India, for a period of six months, from April to September 2023. This study was conducted in medicine wards after due permission from hospital management and prior approval from the institutional ethics committee (ref: JSSMC/IEC/240323/42/NCT/2022-23) dated March 27, 2023.

Inclusion and exclusion criteria

The study participants included all adult patients of more than 18 years of age of both sexes whose samples were sent for blood culture and sensitivity and those who were on empirical antimicrobial therapy during the study period. Pediatric patients and patients admitted and shifted to other wards and ICUs were excluded from the study.

Sample size

Male and female medicine wards in the study setting have a total of 178 beds. Due to various reasons, like bed occupancy rates, the prevalence of seasonal infections, and other factors, a convenience sampling technique was adopted in this study.

Study design

The study was designed in three phases: pre-intervention (two months), intervention (one month), and post-intervention phase (three months). Information on the following variables was collected during the pre-intervention and post-intervention phases: microbiological blood culture investigation sent before administering the first dose of antibiotics; microbiological blood culture investigation sent at least in the first 24 hours of starting antibiotics; microbiological blood culture investigation sent 48 hours after starting antibiotics (only when the patient is not responding to the antibiotic started i.e, those patients who did not show any clinical improvement, having persistent or worsening symptoms, elevated levels of inflammatory markers or with any abnormal radiological findings and no improvement in clinical parameters); microbiological culture investigation not sent; and compliance with hospital antibiotic policy in starting empirical treatment.

During the intervention phase, the investigating team along with the Hospital Infection Control Committee (HICC) members took daily rounds, and emphasis was placed on the importance of microbial culture investigation. All the HCWs, including doctors, nurses, residents, and interns, were emphasized on the importance of sending samples for microbiological culture and sensitivity testing before the initiation of empirical antimicrobial therapy. Also, the pre-intervention data was shown to the concerned healthcare staff as evidence-based information for them to understand the status of culture investigations. Visual info graphs were used to emphasize antimicrobial stewardship practices, including adherence to institutional antibiotic policy.

Statistical analysis

All the descriptive data required for the study, such as sociodemographics, information on the initiation of empirical antimicrobial therapy, type of sample sent for culture, availability of culture sensitivity reports, escalation and de-escalation of antibiotics, and outcome of the therapy, were documented in Microsoft Excel (Microsoft Corp., Redmond, WA), and the descriptive statistics were calculated using IBM SPSS Statistics software for Windows, version 20.0 (IBM Corp., Armonk, NY). The chi-square test and p-value were calculated using the defined software. A p-value <0.10 was considered statistically significant (this is to allow for a higher probability of detecting a true effect, potentially accommodating smaller sample sizes or less stringent requirements in exploratory or preliminary research).

## Results

A total of 256 consecutive patients (120 in the pre-intervention phase and 136 in the post-intervention phase) in the medicine ward on empirical antimicrobial therapy were included in the study. As shown in Table 1, the mean age of patients included in the study during the pre-and post-intervention phases was 47 and 44 years, respectively, with an interquartile range of three. We also noted that during the pre-intervention phase, 28% of the samples were sent before the administration of antibiotics, 39% of the samples were sent within 24 hours of starting empirical antibiotics, 11.7% of samples were sent after 48 hours of antibiotic administration, and in 20.9% of the patients, no culture investigations were sent (Table 2). In the context of the study, during the intervention phase, we conducted individual discussions with the clinical team. The primary reason frequently cited for delaying the submission of patient samples for culture testing beyond 48 hours after the initiation of antibiotics was the lack of patient response to empirical treatment. Additionally, the most prevalent rationale for not requesting culture investigations altogether was attributed to instances of oversight or forgetfulness.

**Table 1:** Sociodemographic characteristics of the patients

Phase	Point prevalence survey	Total patients (n=256)	Mean age	Male and female ratio
Pre-interventional phase	Q1	120	47.93	1.10:1
Post-interventional phase	Q2	136	44.29	1.15:1 256

**Table 2:** Showing compliance of cultures sent before and after antimicrobial therapy in correlation with time duration Q1: pre-intervention phase (two months); Q2: post-intervention phase (three months)

Quarter	Total patients	Total number of patients for whom culture investigation was requested			Total number of patients for whom culture investigation was not requested
		Total samples sent before antibiotics administration	Total samples sent within 24 hours of antibiotics administration	Total samples sent after 48 hours of antibiotic administration	
Q1	120	34 (28%)	47 (39%)	14 (11.7%)	25 (20.9%)
Q2	136	57 (41.9%)	51 (37.5%)	9 (6.6%)	19 (13.9%)
Chi-square test		6.90			
P-value		0.07 (The result is significant at p < 0.10)			

In the post-intervention phase, it was noted that the incidence of utilizing culture investigations increased from 79% in the pre-intervention phase to 86% in the post-intervention phase. Note that the culture sent before starting antimicrobial agents increased from 28% to 41.9% (Table 2).

Sending microbiological cultures before starting antibiotics is crucial to identify the specific pathogen causing an infection and its susceptibility to antibiotics, ensuring that patients receive the most effective and targeted treatment, thus minimizing antibiotic resistance and improving patient outcomes. During the pre-interventional phase, only 28% of the samples were sent before antibiotic administration; in contrast, 41% of the samples were sent during the post-interventional phase. Thirty-nine percent, 11.7%, 37.5%, and 6.6% of samples were sent after 24 and 48 hours of antibiotic administration during the pre-and post-interventional phases, respectively, and are statistically significant. 

In the current study, a statistically significant disparity in culture positivity rates was observed between samples sent prior to the initiation of the first dose of antibiotics (64.7% in pre-intervention phase and 73.68% in post-intervention phase) and those sent within 24 hours (59.27% in pre and 62.7% in post-intervention phase) or 48 hours (35.7% in pre-intervention phase and 22.2% in post-intervention phase) following the commencement of empirical treatment (Table 3).

**Table 3:** Percentage of a better yield of culture positivity among samples sent within the appropriate time duration. Q1: pre-intervention phase (two months); Q2: post-intervention phase (three months)

Quarter	Total samples before administration of antibiotics	Percentage of culture positivity	Total samples sent within 24 hours of administration of antibiotics	Percentage of culture positivity	Total samples sent after 48 hours of administration of antibiotics	Percentage of culture positivity
Q1	34	22 (64.7%)	47	28 (59.57%)	14	5 (35.7%)
Q2	57	42 (73.68%)	51	32 (62.7%)	9	2 (22.2%)
Total	91	64 (70.32%)	98	60 (61.22%)	23	7 (30.43%)

In the context of our study, we conducted an analysis of compliance rates. Our findings indicated a compliance rate of 65.8% during the pre-intervention phase, which subsequently increased to 79.4% in the post-intervention phase (Table 4).

**Table 4:** Percentage compliance with empirical antimicrobial therapy in correlation with existing hospital antibiotic policy Q1: pre-intervention phase (two months); Q2: post-intervention phase (three months)

Quarter	Total patients (n=256)	Percentage of compliance with antibiotic policy
Q1	120	65.8%
Q2	136	79.4%

## Discussion

Antimicrobial stewardship plays a crucial role in promoting the responsible and effective use of antibiotics to combat antibiotic resistance, protect patient safety, and preserve the effectiveness of these vital drugs for future generations. It involves a coordinated effort to optimize antibiotic prescribing, minimize unnecessary use, and ensure the right antibiotic is prescribed at the right dose and duration, benefiting both individual patients and public health.

Antimicrobial stewardship practices are crucial for combating AMR and ensuring optimal patient outcomes, but their implementation may face challenges due to the need for additional resources like personnel and equipment. These upfront costs can act as barriers for those considering adopting ASPs. However, there's been a noticeable shift towards assessing the clinical and economic impact of ASPs through various studies in recent years. This trend reflects a growing recognition of the importance of measuring the effectiveness and cost-effectiveness of these practices. Such evaluations provide valuable insights into the benefits of ASPs, helping to justify the investment in resources and facilitating their implementation and sustainability. Microbiological culture investigation plays a crucial role in implementing AMSP. It is vital for identifying and characterizing infectious microorganisms in clinical specimens, enabling healthcare professionals to diagnose infections, select appropriate treatments, and track antibiotic susceptibility patterns, thus improving patient care and aiding in the management of infectious disease outbreaks [8].

The present study discusses the important issue of how often antimicrobial prescriptions are based on culture results. Similar to our study, Bajpai et al. noted that 43.9% of the patient’s culture samples were not sent before starting antibiotic treatment [11]. Another study done by Garg et al. came out with a similar finding: 41.6% of the patients were treated with antibiotics empirically without sending any microbiology culture investigations [12]. Within the scope of our research, we leveraged the pre-intervention findings to tailor our interventional methodologies.

A systematic review conducted by Dik et al. examined economic evaluations of hospital AMSPs from January 2000 to November 2014 [13]. The review identified 99 studies, predominantly from North America and Europe. These studies assessed various types of stewardship interventions, with "therapy evaluation, review, and/or feedback" being the most frequently evaluated approach, highlighting the common practice of ASP strategies aimed at continuously reviewing and optimizing antimicrobial therapy decisions within hospital settings to improve patient outcomes and combat resistance.

The current data highlight the significant loss of valuable information that occurs when cultures are sampled during antibiotic therapy, potentially compromising the ability to tailor antibiotic treatment effectively. This deficiency poses significant challenges, particularly in optimizing and de-escalating antibiotic therapy, which heavily relies on identifying the specific pathogens causing infection.

We found that patient samples for culture testing are often delayed beyond 48 hours of antibiotic initiation due to a lack of response to empirical treatment. Similarly, overlooking or forgetting to request culture investigations is the most common reason for not initiating them at all. As a unique approach, we also analyzed the timing of the culture sent to understand the correlation with the culture positivity rate. Sending a culture before starting antibiotics helps identify the specific pathogen causing the infection and its susceptibility to antibiotics, allowing for targeted and effective treatment, minimizing the risk of antibiotic resistance, and improving patient outcomes. 

A prospective clinical cohort study of septic patients conducted by Scheer et al. [14] focused on ICU patients diagnosed with sepsis between 2010 and 2017. Specifically, the researchers examined patients who had two or three sets of blood cultures taken at the onset of sepsis. They compared patients whose blood cultures were obtained before antibiotic therapy with those whose samples were taken during antibiotic therapy. The primary comparison was the positivity of blood cultures, which was defined as the presence of a microbiological pathogen. By analyzing these data, the researchers aimed to evaluate whether there was a difference in blood culture positivity between patients whose samples were obtained before antibiotic therapy and those taken during antibiotic therapy. In total, 559 patients with 1,364 blood culture sets at the beginning of sepsis were analyzed. Blood culture positivity was 50.6% (78/154) among patients with sepsis who did not receive antibiotics (i.e., whose blood samples were sent for culture and sensitivity even before the initiation of empirical antimicrobial therapy) and only 27.7% (112/405) in those who were already receiving antibiotics (p <0.001) [14]. Additional challenges to the implementation of recommendations included a lack of emphasis on the link between nursing practices and patient outcomes and anticipated prescriber pushback; hence, efforts to engage nurses in antibiotic stewardship should focus on the nurse's role in improving patient care and outcomes, an approach that has succeeded in minimizing healthcare-associated infections. Healthcare providers across the board are increasingly acknowledging the vital role of AMSPs within healthcare institutions. These programs are seen as critical contributors to maintaining the effectiveness of antimicrobial agents in treating infections over the long term. While the number of studies on this topic may be relatively limited, evidence compiled from meta-analyses indicates that clinical outcomes tend to be either improved or at least comparable for patients when AMSPs are implemented. This suggests that AMSPs are not only beneficial but may actually enhance patient care and treatment effectiveness, reinforcing their importance in modern healthcare settings. [15]. Hence, during the interventional phase, daily rounds were taken by AMSP members, and emphasis was placed on the importance of microbial culture investigation.

The pre-intervention data were presented to healthcare staff as evidence-based information, utilizing visual infographics to highlight AMSP practices, including adherence to institutional antibiotic policies. This interventional study highlights the effectiveness of an AMSP that can emphasize the importance of sending cultures before empirical antimicrobial therapy and antibiotic prescribing practices, which target the role of structural monitoring in achieving positive outcomes and combating the growing problem of antimicrobial resistance. Compliance with an institutional antibiotic policy is essential to optimize the use of antibiotics, reduce the risk of antibiotic resistance, and improve patient safety by ensuring that healthcare providers follow evidence-based guidelines for prescribing antibiotics, promoting judicious use while minimizing the potential for adverse effects and treatment failures.

## Conclusions

In conclusion, the concept of a "culture of cultures" represents a promising approach to enhancing diagnostic stewardship in healthcare. This innovative idea promotes integrating diverse cultural perspectives, beliefs, and practices into the diagnostic process to improve patient outcomes and foster holistic, patient-centered care. While this study offers valuable insights into judicious antimicrobial utilization, thereby enhancing patient care and combating antibiotic resistance in hospitals, it does have limitations. Specifically, the study was conducted in a single unit of a tertiary care hospital and had a limited sample size.

Despite these limitations, the implemented intervention showed statistically significant results, suggesting its potential for broader application across different hospital departments and healthcare settings. This research marks a crucial step toward establishing a culture of evidence-based investigation in treating infectious diseases, aiming to reduce unnecessary antimicrobial use effectively.
